# Surgical Technique for Treating Focal Osteochondral Knee Lesions Using an Aragonite‐Based Biphasic Scaffold

**DOI:** 10.1002/atn2.70033

**Published:** 2026-04-30

**Authors:** Eric Wang, Alexandra M. Cancio‐Bello, Katelyn T. Koschmeder, Bryn Berens, Anikar Chhabra

**Affiliations:** ^1^ Mayo Clinic Alix School of Medicine Mayo Clinic Arizona Phoenix Arizona U.S.A.; ^2^ Department of Orthopedic Surgery Mayo Clinic Phoenix Arizona U.S.A.

## Abstract

Osteochondral lesions of the knee, particularly in patients with early osteoarthritic changes, remain a clinical challenge. The Agili‐C implant (CartiHeal, Smith & Nephew), an aragonite‐based osteochondral scaffold, offers a single‐stage, off‐the‐shelf solution designed to regenerate both cartilage and subchondral bone. Unlike cell‐based therapies, Agili‐C eliminates the need for cell harvesting, culturing, or bioreactors, and avoids the morbidity associated with autografts and allografts. Recent multicenter randomized controlled trials have showed superior functional outcomes and pain reduction with Agili‐C compared to microfracture and debridement, even in patients with early osteoarthritis (Kellgren‐Lawrence grades 0‐3). This Technical Note describes the surgical technique for Agili‐C implantation and highlights key technical pearls to optimize outcomes.

## Graphical abstract

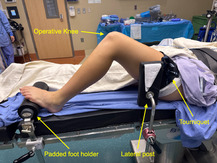

**VIDEO 1:** Disclosures are presented (00:18). Patient is positioned supine with a tourniquet on the proximal thigh; the leg is prepped and draped in standard fashion (00:23). Arthroscopy begins through anteromedial and anterolateral portals with diagnostic evaluation and synovectomy to improve visualization; all compartments are assessed (00:42). The osteochondritis dissecans (OCD) lesion in the medial femoral condyle is probed and found unstable, with underlying sclerotic bone and osteophytes indicating chronic pathology (00:59). Arthroscopic instruments are removed, and a medial parapatellar arthrotomy is performed for open exposure of the lesion (01:07). Loose fibrocartilage is debrided. and the lesion, measuring 20 × 10 mm, is cleaned with a curette (01:22). Two 7.5 mm Agili‐C implants are selected and placed with 3 to 5 mm spacing and 2 to 3 mm from healthy cartilage margins (02:04). Using CartiHeal instrumentation, a K‐wire is drilled perpendicular to the joint surface, followed by cannulated drilling and refinement with a hand reamer until the laser line disappears beneath the subchondral surface (02:22). Defect walls are smoothed, lesion geometry finalized with a shaper, and the site is irrigated for implant seating (03:03). The Agili‐C implant is inserted and tamped into place with the rubber‐tipped impactor, perpendicular to the joint surface (03:22). Implant is recessed 2 to 6 mm below the subchondral bone; if proud, redrilling is required for a larger implant (03:33). For multiple lesions, the process is repeated with proper spacing; arthroscopic and gross inspection confirm stable positioning of both implants (03:50). Our sample rehabilitation protocol is presented, noting that rehab is individualized based on surgeon and patient specifics (04:08). Video content can be viewed at https://doi.org/10.1002/atn2.70033.

Osteochondral defects of the knee are a common source of pain, swelling, and functional limitation, particularly in young, active individuals and patients with early degenerative changes.^1^ Treatment options such as microfracture, osteochondral autograft transfer, and autologous chondrocyte implantation have shown variable success, often limited by donor site morbidity, graft availability, and the need for complex, multistage procedures.^2^, ^3^, ^4^ Given these challenges, there is a need for an effective, less invasive, and technically straightforward solution.

The Agili‐C implant (CartiHeal, Smith & Nephew, Andover, MA, USA) is a single‐stage, off‐the‐shelf, aragonite‐based scaffold designed to support regeneration of both cartilage and subchondral bone.^5^ In a landmark multicenter randomized controlled trial enrolling 251 patients aged 21 to 75 years across 26 medical centers, Agili‐C showed superior clinical and radiographic outcomes compared to microfracture and debridement, even in patients with early osteoarthritis (Kellgren‐Lawrence grades 0‐3).^6^ The purpose of this Technical Note is to describe our approach to Agili‐C implantation for osteochondral lesions of the knee, including key steps in lesion preparation, graft placement, and intraoperative technique.

## SURGICAL TECHNIQUE

### Preoperative Assessment

Evaluation begins with a focused history and physical examination, including assessment of pain, swelling, mechanical symptoms (e.g., locking or catching), and prior treatments such as physical therapy, injections, or surgery. The physical exam should assess for joint line tenderness, effusion, and mechanical signs, while screening for malalignment, ligamentous instability, and meniscal pathology.

Radiographic evaluation includes weight‐bearing anteroposterior, 30° posteroanterior, lateral, and patellofemoral views to assess joint space narrowing, osteophyte formation, and Kellgren‐Lawrence grade. Long‐leg alignment films are obtained to evaluate mechanical axis deviation of the limb. Magnetic resonance imaging is used to characterize osteochondral lesion size, depth, and location and to assess surrounding cartilage, subchondral bone, and bone marrow edema. Computed tomography may be used selectively for surgical planning or to evaluate subchondral bone quality (Table 1).

**TABLE 1:** Indications and Contraindications for Agili‐C Osteochondral Scaffold Implantation

Indications	Contraindications
• Symptomatic focal chondral or osteochondral defect of the femoral condyle or trochlea	• Lesions on the tibial plateau or patella
• ICRS grade ≥III lesions	• KL grade IV osteoarthritis
• Age 18 to 75 yr	• Avascular necrosis at the lesion site
• Lesion size between 1 to 7 cm^2^ (up to three discrete lesions)	• Active joint or systemic infection
• KL grade 0 to III osteoarthritis	• Uncontained lesions not bordered by vital cartilage or lacking ≥2 mm subchondral bone rim
• Mechanical alignment within 5° of neutral (or correctable at surgery)	• Subchondral bone defects or cysts >8 mm deep
• BMI < 35	• Malalignment >5° not corrected at time of surgery
	• BMI > 35
	• Skeletal immaturity

BMI, body mass index; ICRS, International Cartilage Repair Society; KL, Kellgren‐Lawrence; yr, year.

### Patient Positioning

**FIGURE 1:**
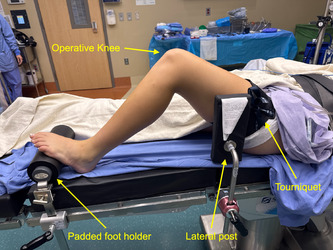
Example of a patient positioned supine with the left operative leg prepped and draped to allow full range of motion. Standard anteromedial and anterolateral arthroscopic portals are established for diagnostic evaluation of the lesion and joint.

A video showing this technique is provided (Video 1). Under general anesthesia, the patient is placed supine on a standard operating table. The operative leg is positioned to allow full flexion and extension of the knee, with the foot and thigh stabilized using a padded foot holder and a lateral post. If needed, an assistant may help maintain stability during high‐force steps such as drilling. A proximal thigh tourniquet is applied, and the knee and distal thigh are prepped and draped in standard sterile fashion (Figure 1).

### Approach, Exposure, and Planning

**FIGURE 2:**
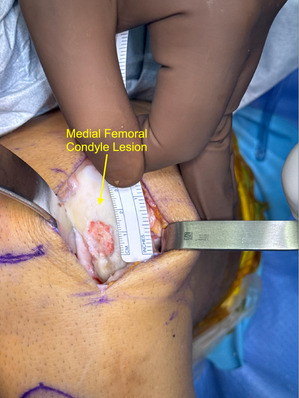
Patient supine, right knee. Mini‐arthrotomy performed to expose the lesion. Unstable cartilage and loose bodies are removed, and lesion dimensions are measured to guide implant selection. Margins are confirmed to have stable cartilage and ≥2 mm of subchondral bone rim.

**FIGURE 3:**
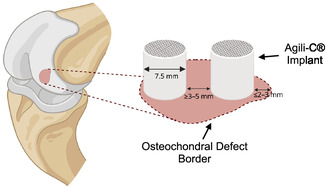
Implant number and spacing are planned based on lesion size and configuration. A minimum 3 to 5 mm bone bridge is maintained between implants, with placement ideally within 2 to 3 mm of healthy cartilage margins.^7^

First, diagnostic arthroscopy is completed to confirm osteochondral lesion location and joint status. Next, a mini‐arthrotomy is performed slightly medial to the midline. Unstable cartilage and loose bodies are removed to expose the defect. The lesion must be well‐contained—surrounded by viable cartilage and a circumferential rim of subchondral bone ≥2 mm—to ensure proper implant seating (Figure 2). Once the lesion is confirmed, the dimensions are measured to determine number of implants and size (Figure 3).^7^ Implants are shelf‐stable and available in 7.5 mm (standard), 10 mm (rescue), and 12.5 mm (rescue) diameters. Ideal placement should maintain ≥3 to 5 mm of bone bridge between implants and ≤2 to 3 mm between implant edge and lesion margin.

### Implant Site Preparation

**FIGURE 4:**
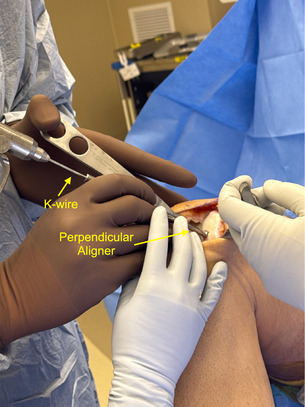
Patient supine, right knee. K‐wire inserted perpendicularly through the center of the lesion using a perpendicular aligner to guide trajectory and depth.

**FIGURE 5:**
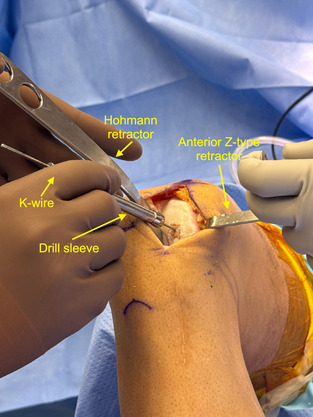
Patient supine, right knee. Drill sleeve placed over the K‐wire following removal of the perpendicular aligner, preparing for cannulated drilling.

**FIGURE 6:**
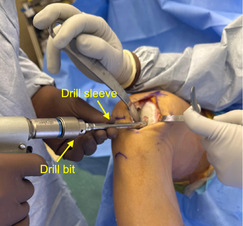
Patient supine, right knee. Cannulated drill advanced over the K‐wire to the predetermined depth using a hard stop, ensuring controlled reaming of the defect.

**FIGURE 7:**
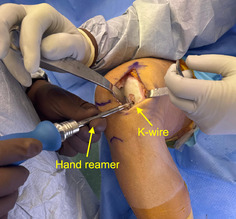
Patient supine, right knee. Hand reamer advanced over the K‐wire and manually rotated until the laser line disappears beneath the subchondral surface. Firm pressure is applied to smooth defect walls and ensure unobstructed implant insertion.

**FIGURE 8:**
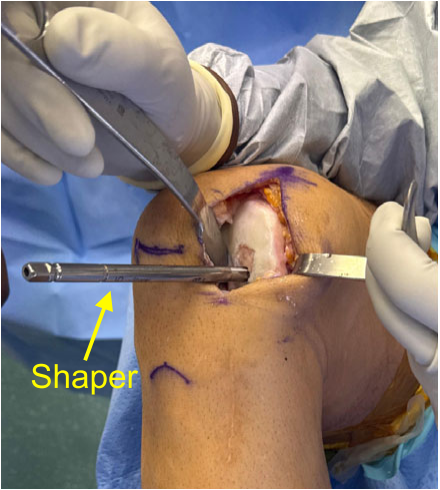
Patient supine, right knee. Lesion shaper used to finalize hole dimensions and as secondary step to confirm appropriate depth and cylindrical conformity of the defect

**FIGURE 9:**
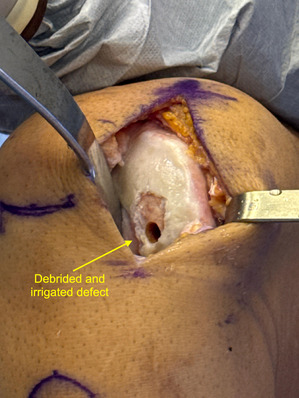
Patient supine, right knee. Defect edges debrided using a cartilage cutter and scalpel following K‐wire removal. Thorough irrigation performed to remove debris and optimize implant seating.

The lesion center is marked, and a perpendicular alignment guide is positioned. A K‐wire is inserted perpendicular to the articular surface (Figure 4), followed by a cannulated drill sleeve (Figure 5). Drilling is performed to the hard stop (Figure 6) and then refined with a hand reamer until the laser mark is just below the cartilage surface (Figure 7). A lesion shaper is used to finalize depth and conformity (Figure 8). The K‐wire is removed, and the site is irrigated. Residual debris is cleared using a cartilage cutter and scalpel (Figure 9). Thorough irrigation is critical, as debris may prevent proper implant seating and necessitate redrilling for a larger implant.

### Final Agili‐C Implantation

**FIGURE 10:**
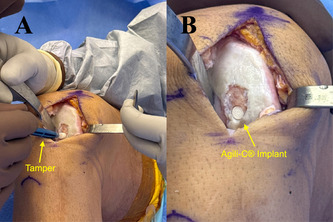
Patient supine, right knee. Final implant insertion and seating. (A) The Agili‐C implant is gently inserted into the prepared defect using the provided rubber‐tipped blue tamp, with the holes facing the surgeon and alignment maintained perpendicular to the joint surface. (B) Final implant shown fully seated flush with the subchondral bone and recessed beneath the cartilage surface. Acceptable implant positioning ranges from just below to 4 mm below the subchondral bone; if proud, the site must be redrilled for a larger implant, as removal is not possible once inserted.

**FIGURE 11:**
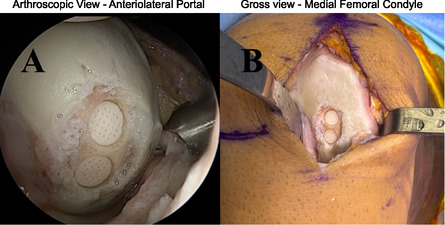
Patient supine, right knee. Final implant positioning and confirmation. (A) Arthroscopic view confirming that both Agili‐C implants are seated flush with the surrounding cartilage surface. (B) Gross visualization showing two implants with appropriate spacing (≥3‐5 mm) and stable positioning within the prepared defects.

The implant is manually inserted with orientation holes facing the surgeon and gently tamped into place using the blue‐handled impactor. It must be inserted perpendicular to the articular surface to avoid fracture. A tactile “click” confirms seating. The implant should rest slightly recessed, ideally 0.5 mm below the subchondral bone (Figure 10A,B). If proud, the site must be redrilled, as the implant cannot be removed once deployed. For multiple lesions, repeat the steps, ensuring ≥3 to 5 mm bone bridges and ≤2 to 3 mm proximity to healthy cartilage. Final inspection confirms implant position and stability (Figure 11). The joint is irrigated and closed in layers with a compressive dressing applied. Pearls and pitfalls of the procedure are summarized in Table 2.

**TABLE 2:** Pearls and Pitfalls of Agili‐C Implantation for Focal Osteochondral Lesions of the Knee

Pearls	Pitfalls
• Preoperative imaging should include MRI and long‐leg alignment radiographs to assess lesion containment, subchondral bone quality, and mechanical axis	• Inadequate imaging may fail to identify contraindications, including uncontained lesions or advanced KL grade changes
• Perform diagnostic arthroscopy to confirm lesion location and rule out contraindications	• Suboptimal limb positioning can limit exposure and lead to angulated drilling or uneven implant seating
• Position the leg to allow full flexion and extension; use a padded foot holder or lateral post to stabilize the limb during drilling	• Excessive debridement may remove viable cartilage, reducing scaffold support and increasing the risk of failure
• Preserve stable cartilage margins during debridement; remove only unstable or damaged tissue to support scaffold integration	• Improper implant spacing or alignment can result in fracture, instability, or poor integration
• Maintain a bone bridge of ≥3 to 5 mm between implants, ensuring each site is surrounded by stable cartilage and a circumferential rim of subchondral bone at least 2 mm thick to support proper implant seating	• Implantation in patients with diffuse cartilage loss, KL grade IV changes, or uncorrected malalignment may lead to early failure
• Irrigate thoroughly and prepare the site carefully to remove debris and ensure flush or slightly recessed implant seating	

KL, Kellgren‐Lawrence; MRI, magnetic resonance imaging.

### Postoperative Protocol and Rehabilitation

Postoperative care follows principles similar to other cartilage restoration procedures. Patients should remain toe‐touch weightbearing with crutches for the first 4 weeks, followed by progression to full weightbearing for 2 additional weeks. Immediate isometric quadriceps exercises are initiated, along with immediate active and passive range of motion. Per surgeon preference, a continuous passive motion machine can be used for motion assistance in the early postoperative period. Submersion in water and hydrotherapy may begin after suture removal and complete wound healing. Progression to functional rehabilitation is individualized based on lesion characteristics and patient factors. A return to full activities is expected at 6 months postoperatively.

## DISCUSSION

This Technical Note describes a standardized approach for Agili‐C implantation in the treatment of focal osteochondral lesions of the knee. The technique emphasizes critical surgical principles, including accurate lesion assessment, preservation of stable cartilage margins, and secure, contained implantation, to support graft stability and biologic integration.

The rationale for using a biphasic scaffold that targets both cartilage and subchondral bone is grounded in their well‐established interplay.^8^ Subchondral bone damage contributes to joint degeneration, particularly in early osteoarthritis and post‐traumatic settings.^9^, ^10^ The aragonite‐based composition of Agili‐C has showed osteoconductive and chondrogenic properties in preclinical studies, supporting cell adhesion and osteochondral differentiation.^11^, ^12^ Clinical data on Agili‐C continues to grow. In 2023, a multicenter randomized controlled trial significantly outperformed microfracture and debridement, with superior clinical and radiographic outcomes sustained through 2 years.^6^ At final follow‐up, the implant group showed double the mean improvement in the Knee injury and Osteoarthritis Outcome Score compared to controls, with a responder rate of 77.8% versus 33.6%. Radiographically, 88.5% of Agili‐C patients achieved greater than 75% defect fill, and the failure rate was notably lower (7.2% vs 21.4%). These outcomes were consistent across both focal and early degenerative lesions, including patients treated with multiple implants. Additional cohort studies have reported low complication rates and favorable outcomes in cases involving multiple Agili‐C implants.^13^, ^14^, ^15^, ^16^, ^17^ Compared to earlier‐generation scaffolds, Agili‐C addresses several limitations. TruFit was withdrawn due to poor incorporation, while MaioRegen has shown mid‐term success in select populations.^18^, ^19^ No direct comparative trials exist, though a 2021 systematic review found generally favorable outcomes for both MaioRegen and Agili‐C despite study heterogeneity.^20^

From a surgical standpoint, Agili‐C offers several practical and biologically relevant advantages. Its off‐the‐shelf, cell‐free design enables single‐stage restoration of cartilage and subchondral bone without donor tissue, cell harvesting, or lab‐based expansion. The scaffold promotes chondrocyte migration and formation of hyaline‐like matrix deposition, while its osteoconductive properties promote subchondral bone regeneration. These biological and practical features make it suitable for a broad range of patients, including those with mild to moderate osteoarthritis or multifocal lesions. Additionally, the implantation technique is relatively straightforward and does not require fixation in well‐contained defects, reducing surgical complexity and morbidity compared to autograft or two‐stage procedures.

However, several limitations must be acknowledged. Although early clinical outcomes are encouraging, most published studies are case series or nonrandomized cohorts, and long‐term data beyond 5 years remain limited. One study has reported sustained improvements at a mean follow‐up of 6.5 years, but larger prospective trials are needed.^16^ Moreover, Agili‐C may be less suitable for large, deep, or uncontained defects, particularly those that exceed 7 cm^2^ in area or 8 mm in depth, where stable containment is challenging. Inadequate lesion preparation or improper implant spacing may result in suboptimal fill, early graft failure, or compromised joint congruity. Additionally, Agili‐C has not been directly compared to established techniques such as matrix‐induced autologous chondrocyte implantation and osteochondral allograft transplantation, which have showed robust long‐term results in complex lesions. While the procedure avoids graft harvesting and is relatively simple, success remains dependent on appropriate patient selection and careful implant planning. Table 3 summarizes the advantages and disadvantages of Agili‐C implantation.

**TABLE 3:** Advantages and Disadvantages of Agili‐C for Focal Osteochondral Lesions of the Knee

Advantages	Disadvantages
• A single‐stage, off‐the‐shelf procedure that addresses both cartilage and subchondral bone without the need for cell harvesting or donor tissue	• May be less suitable for large, deep, or uncontained lesions where achieving stable containment is difficult
• The biphasic scaffold supports biologic integration and osteochondral repair through chondrogenic and osteoconductive properties	• Inadequate lesion preparation or over‐aggressive debridement can compromise scaffold support and integration
• Can be used in a range of lesion sizes and locations, including multifocal defects	• Improper implant spacing or depth may result in joint surface incongruity or early mechanical failure
• Implantation is technically straightforward and does not require fixation in contained lesions.	
• Avoids the morbidity and logistical complexity associated with autograft or allograft procedures	

In summary, Agili‐C represents a promising single‐stage solution for osteochondral repair, particularly in patients for whom traditional techniques are suboptimal. Its ease of use, biologic potential, and favorable early outcomes support its growing role in cartilage restoration. However, further long‐term studies and comparative trials are essential to fully define its role in the evolving treatment landscape.

## DISCLOSURES

The authors (E.W., A.M.C.‐B., K.T.K., B.B., and A.C.) declare that they have no known competing financial interests or personal relationships that could have appeared to influence the work reported in this article.
